# Hours for Recreation

**Published:** 1870-01

**Authors:** 


### HOURS FOR RECREATION.

No one should work or study hard within half-an-hour of a regular meal.

The time for profitable and efficient study is in the early part of the day, certainly before noon, because sleep is the rest of the brain; and while it is thus resting, nature is depositing new particles upon which it is to feed the next day. To engage in severe study in the after-part of the day leads to an injurious exhaustion, over-straining of the brain, and is as unwise as for a man to engage in hard work at sundown, when the strength of the body has already been used up in the ordinary occupations. The best time for recreation, as to body or brain, is the after-part of the day, when the main business of life has been attended to; thus resting the working faculties, while exercising those which have been idle; thus giving occupation each day for the whole man.

